# Adaptability of the ubiquitin-proteasome system to proteolytic and folding stressors

**DOI:** 10.1083/jcb.201912041

**Published:** 2020-12-31

**Authors:** Jeremy J. Work, Onn Brandman

**Affiliations:** Department of Biochemistry, Stanford University, Stanford, CA

## Abstract

Aging, disease, and environmental stressors are associated with failures in the ubiquitin-proteasome system (UPS), yet a quantitative understanding of how stressors affect the proteome and how the UPS responds is lacking. Here we assessed UPS performance and adaptability in yeast under stressors using quantitative measurements of misfolded substrate stability and stress-dependent UPS regulation by the transcription factor Rpn4. We found that impairing degradation rates (proteolytic stress) and generating misfolded proteins (folding stress) elicited distinct effects on the proteome and on UPS adaptation. Folding stressors stabilized proteins via aggregation rather than overburdening the proteasome, as occurred under proteolytic stress. Still, the UPS productively adapted to both stressors using separate mechanisms: proteolytic stressors caused Rpn4 stabilization while folding stressors increased *RPN4* transcription. In some cases, adaptation completely prevented loss of UPS substrate degradation. Our work reveals the distinct effects of proteotoxic stressors and the versatility of cells in adapting the UPS.

## Teaser

Work and Brandman measure how the ubiquitin-proteasome system (UPS) adapts to different types of stressors. They find that the UPS can adapt almost perfectly to stress conditions, even those that stabilize misfolded proteins through aggregation rather than increasing UPS substrate load.

## Introduction

The ubiquitin-proteasome system (UPS) is the primary route for the disposal of defective proteins in eukaryotic cells (Hershko et al., 1983, 1984; Lecker et al., 2006). Aging, genetic mutations, and environmental changes challenge the UPS and can lead to accumulation of defective proteins (“proteotoxic stress”), which is a hallmark of many neurodegenerative diseases, including Alzheimer’s disease, Parkinson’s disease, Huntington’s disease, and amyotrophic lateral sclerosis (Labbadia and Morimoto, 2015; Sweeney et al., 2017; Klaips et al., 2018). Characterizing the performance and adaptability of the UPS in clearing defective proteins under proteotoxic stressors is thus likely to aid in understanding numerous diseases.

In the UPS, ubiquitin ligases modify selected proteins with polyubiquitin chains that target them for degradation by the 26S proteasome, a 2.5-MD protein complex composed of 33 unique subunits (Voges et al., 1999). To simultaneously and stoichiometrically drive the expression of dozens of proteasomal components along with other UPS-related genes, eukaryotes have evolved master transcriptional regulators that target all proteasome genes, as well as ubiquitin, ubiquitin ligases, and extrinsic proteasome factors (Mannhaupt et al., 1999; Lundgren et al., 2003; Meiners et al., 2003; Kraft et al., 2006; Xu et al., 2008; Sato et al., 2009; Radhakrishnan et al., 2010). In budding yeast, this master regulation occurs via the transcription factor Rpn4 (Xie and Varshavsky, 2001).

To adapt the expression of UPS components based on cellular needs, cells regulate Rpn4 levels via multiple stress-sensitive mechanisms. These include proteasomal degradation of Rpn4 via two encoded degradation signals (degrons) that target it to the proteasome: one ubiquitin-independent signal at the N-terminus, and one signal recognized by the E3 ubiquitin ligase Ubr2 (Ha et al., 2012; Wang et al., 2004). Due to these degrons, Rpn4 has a short half-life of 2 min and will therefore quickly accumulate if the proteasome is impaired (Xie and Varshavsky, 2001). Additionally, *RPN4* is transcriptionally regulated by several stress-sensitive transcription factors, including Yap1, a responder to oxidative stress; Pdr1/3, the drivers of the pleiotropic drug resistance response; and Hsf1, the driver of the heat shock response (HSR; Hahn et al., 2006; Ma and Liu, 2010; Temple et al., 2005; Moye-Rowley, 2003). We term the collective, stress-responsive transcriptional regulation of the UPS through Rpn4 the proteasome stress response (PSR), analogous to terminology used to describe other transcriptional stress responses like the HSR.

**Figure 1.**
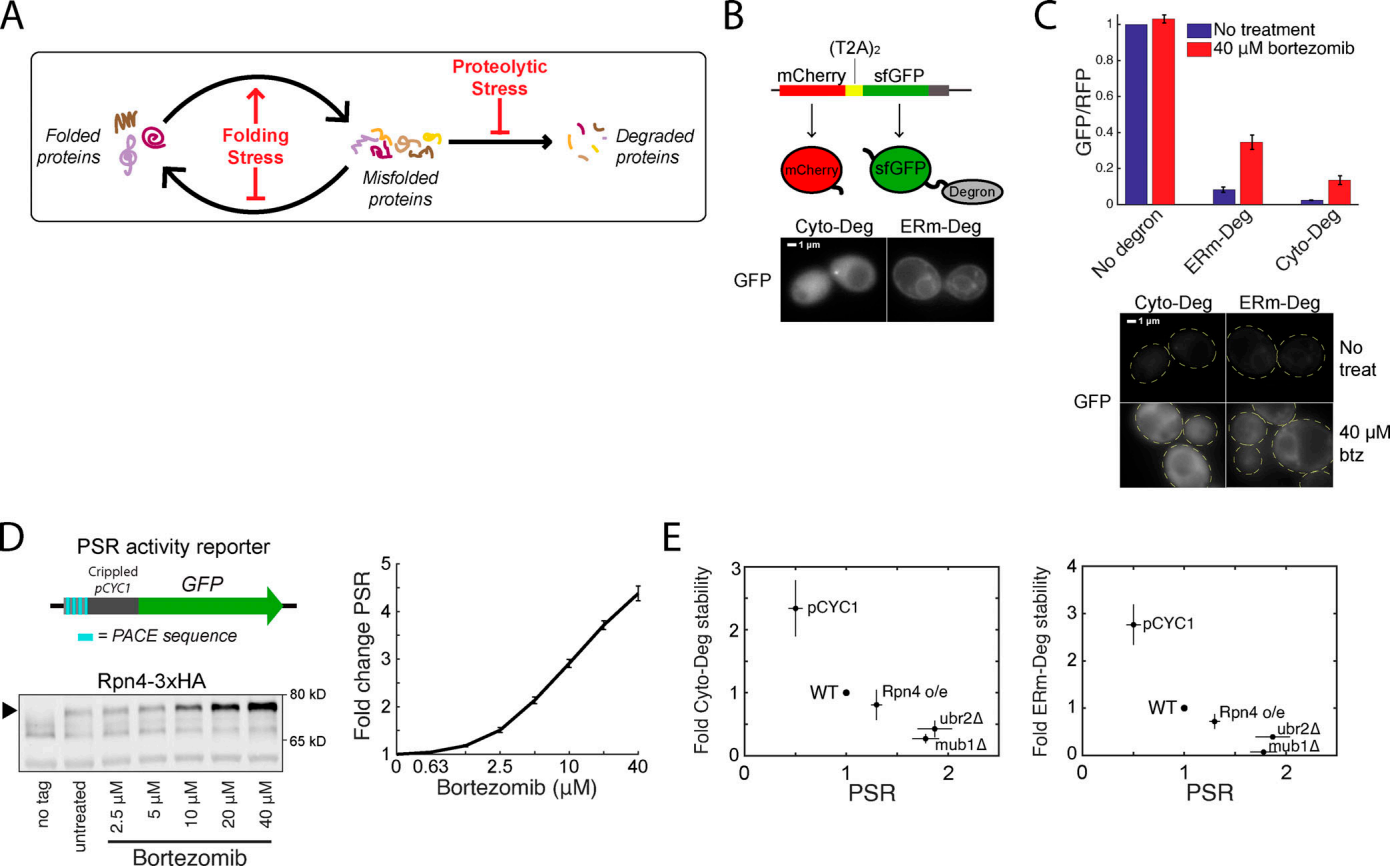
**Clearance of defective proteins scales with the PSR.** **(A)** Diagram of protein folding and degradation, and the effect of folding and proteolytic stress. **(B)** Top: Schematic of T2A system for controlled expression of degron reporters. Bottom: GFP localization of Cyto-Deg and ERm-Deg in normal conditions. **(C)** Top: Mean RFP-normalized GFP fluorescence of Cyto-Deg, ERm-Deg, and a no degron control with either 0 µM (blue bars) or 40 µM (red bars) bortezomib. Bottom: GFP localization of Cyto-Deg and ERm-Deg in either 0 µM or 40 μM bortezomib conditions. Cells are outlined in yellow dashed lines. **(D)** Top left: Schematic of PSR activity reporter. Bottom left: Immunoblot of HA-tagged endogenous Rpn4 under a serial titration of bortezomib. Arrow indicates the position of Rpn4-3xHA. Right: Mean forward scatter normalized GFP of PSR activity reporter under a serial titration of bortezomib. Units are fold change from no treatment. **(E)** Plots of fold degron stability (left: Cyto-Deg; right: ERm-Deg) versus fold PSR upon modifying Rpn4 levels through deletion of *UBR2* or *MUB1*, replacement of the endogenous *RPN4* promoter with *pCYC1*, or expression of a second copy of *RPN4* from a plasmid. Error bars denote standard error for *n* = 3 biological replicates. btz, bortezomib; PACE, proteasome-associated control element; treat, treatment.

While the PSR has been demonstrated to respond to proteotoxic stressors (Xie and Varshavsky, 2001; Wang et al., 2010; Schmidt et al., 2019), quantification of its effectiveness at combating such stressors and the relative contributions of its distinct activation mechanisms have not been investigated in diverse proteotoxic conditions. Two ways stressors may increase levels of misfolded proteins are to (1) cause proteins to misfold or obstruct their folding (“folding stress”), or (2) impair degradation rates of misfolded proteins (“proteolytic stress”; Fig. 1 A). A naive expectation is that folding and proteolytic stressors have overlapping effects on the proteome and UPS. For example, misfolded proteins generated by a folding stressor may become targeted to the proteasome, increasing competition between proteasome substrates and thereby lowering degradation rates for each substrate (i.e., a folding stressor leading indirectly to proteolytic stress). Conversely, UPS substrates that are stabilized by a proteolytic stressor may potentiate the misfolding of other proteins (i.e., a proteolytic stressor indirectly leading to folding stress), as has been observed when expression of one misfolded protein causes others to misfold (Satyal et al., 2000; Gidalevitz et al., 2006, 2009). However, these hypotheses have not yet been quantitatively evaluated. Furthermore, it is unknown if activation of the PSR fully neutralizes proteotoxic challenges (“perfect adaptation”), if cells accumulate defective proteins in spite of PSR activation (“partial adaptation”), if cells overreact to these challenges (“overadaptation”), and whether cellular responses are distinct for proteolytic and folding stressors.

We systematically characterized the performance and adaptability of the UPS under diverse stress conditions in yeast using quantitative measurements of UPS performance (measured by the stability of misfolded reporter substrates) and PSR-mediated adaptation (transcriptional activation of Rpn4 target genes as measured by a synthetic reporter). We unexpectedly found that proteolytic and protein folding stressors generally stabilized misfolded proteins and activated the PSR through separate, nonoverlapping mechanisms. Proteasomal inhibition (a proteolytic stressor) blocked degradation of misfolded proteins and stabilized Rpn4 without increasing *RPN4* transcription. By contrast, the addition of the amino acid analogues canavanine and 2-azetidine-2-carboxylic acid (AZC; folding stressors) caused aggregation of misfolded proteins rather than their targeting to the proteasome, yet still activated the PSR by driving transcriptional activation of *RPN4* without increasing Rpn4 stability. The PSR productively responded to both proteolytic and protein folding stressors despite their different underlying mechanisms for increasing misfolded protein levels. In both cases, this included perfect or near-perfect adaptation. Our work reveals the adaptability of the UPS and provides a framework to quantitatively understand how cells regulate the UPS in response to proteotoxicity and disease-causing states.

## Results

### Clearance of defective proteins scales with the PSR

To investigate the UPS under stress conditions, we built quantitative reporters that measure the performance of the UPS and the activity of the cell’s primary transcriptional effector of the UPS, the PSR. Because a major role of the UPS is to identify and degrade defective proteins, we defined “UPS performance” as the ability to degrade reporter proteins containing constitutively misfolded domains. We designed two UPS substrates, a cytosol-localized degron (Cyto-Deg) and an ER membrane–localized degron (ERm-Deg), consisting of superfolder green fluorescent protein (sfGFP) fused to a degron sequence featuring a cluster of hydrophobic residues (Maurer et al., 2016; Fig. 1 B). Cyto-Deg localizes to the cytosol and is dependent on Hsp70 for degradation (Maurer et al., 2016). ERm-Deg localizes to the ER membrane—likely because its C-terminal degron is also an ER targeting signal—and is ubiquitylated by the ER membrane–localized ubiquitin ligase Doa10 (Maurer et al., 2016). Doa10 is part of the ER-associated degradation pathway for cytosolic domains (ERAD-C), which targets ER transmembrane proteins with a misfolded domain in the cytosol (Ruggiano et al., 2014). Thus, expression of both Cyto-Deg and ERm-Deg leads to misfolded protein domains in the cytosol, but they are efficiently degraded by the proteasome via separate pathways. Evaluating the stability of both degrons therefore informs on the state of the proteasome, the shared feature in their degradation. To control for protein synthesis rate, we expressed a red fluorescent protein (mCherry) upstream of the sfGFP-degron fusion, separated by two tandem T2A peptide-skipping sequences (Donnelly et al., 2001; Szymczak and Vignali, 2005). mCherry and the sfGFP-degron are synthesized stoichiometrically, but mCherry is detached during translation and escapes UPS targeting. The sfGFP/mCherry ratio is therefore proportional to the stability of the degron-fused protein, where a high ratio indicates high stability and a low ratio indicates low stability. Cyto-Deg and ERm-Deg were both capable of reporting on UPS performance, as evidenced by an increase in the sfGFP/mCherry ratio upon treatment with a 40-µM dose of the proteasome-inhibiting drug bortezomib and increased GFP fluorescence by microscopy (Fig. 1 C).

To measure the PSR, we built a synthetic promoter specifically sensitive to changes in the PSR. The PSR is driven by the binding of Rpn4 to a DNA motif called the proteasome-associated control element, which is found in the promoters of all proteasomal subunits and many proteasome-associated factors (Mannhaupt et al., 1999; Shirozu et al., 2015). The PSR reporter features four tandem copies of the proteasome-associated control element sequence along with a minimal promoter to drive expression of sfGFP (Fig. 1 D). We validated the PSR reporter’s sensitivity by inhibiting the proteasome with bortezomib and observing a monotonic increase in GFP expression in response (Fig. 1 D). This increase corresponded to increased levels of hemagglutinin-tagged endogenous Rpn4 (Fig. 1 D).

We first used our reporters to evaluate how modulating the PSR affects UPS performance in unstressed conditions by altering the constitutive levels of Rpn4. To reduce Rpn4 levels, we replaced the endogenous *RPN4* promoter with a weaker promoter (*pCYC1*). To increase Rpn4 levels, we deleted factors necessary for Rpn4 degradation (Ubr2 or Mub1; Ju et al., 2008; Wang et al., 2004), or expressed a second copy of *RPN4* from a single-copy plasmid. PSR activity was inversely correlated with Cyto-Deg and ERm-Deg stability, demonstrating that PSR activity is tightly coupled to UPS performance (Fig. 1 E).

### Proteotoxic stressors elicit multiple adaptive regimes

**Figure 2.**
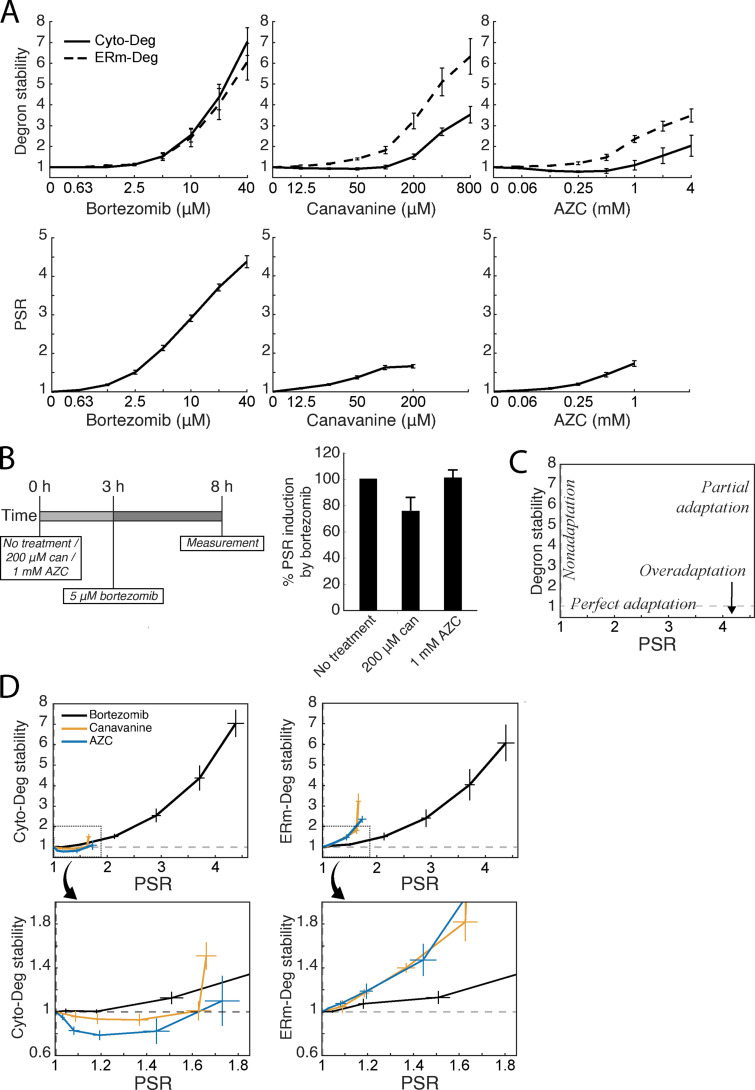
**Proteotoxic stressors elicit multiple adaptive regimes. (A) **Measurements of fold degron stability (top row, solid: Cyto-Deg; dotted: ERm-Deg) and fold PSR activity (bottom row) in titrations of bortezomib, canavanine, or AZC. **(B)** Measurement of PSR activity (right) after treatment with 5 µM bortezomib and either 200 µM canavanine, 1 mM AZC, or no additional treatment, at the noted time points (left). **(C)** Schematic of adaptive regimes as a function of degron stability and PSR activity. **(D) **Plots of fold degron stability (left: Cyto-Deg; right: ERm-Deg) versus fold PSR activity for the titrations in A to reveal the adaptive regime for each stressor. The boxed regions in the upper plots are enlarged in the lower plots. Error bars denote standard error for *n* ≥ 3 biological replicates. can, canavanine.

To understand the adaptive potential of the UPS, we investigated the role of the PSR in clearing defective proteins during proteotoxic stress. We achieved this by measuring the PSR and degron stability in cells after 5 h treatment with three proteotoxic compounds: bortezomib, canavanine (an arginine analogue), and AZC (a proline analogue). Bortezomib directly inhibits the proteasome to cause proteolytic stress. Canavanine and AZC directly disrupt protein folding when incorporated into newly synthesized proteins, causing folding stress (Rodgers and Shiozawa, 2008). By measuring cellular responses after 5 h, we aimed to capture the system after adaptive mechanisms had taken effect and reporters levels were at or approached steady-state values. At the highest concentrations tested, all three stressors increased the stability of both Cyto-Deg and ERm-Deg and induced the PSR, consistent with their proteotoxicity (Fig. 2 A). We were concerned that high doses of canavanine and AZC would directly disrupt PSR reporter inducibility, so we tested its ability to induce via bortezomib after pretreatment with canavanine or AZC. We accordingly limited the concentrations of canavanine and AZC to a range in which the PSR reporter remained comparably inducible by 5 µM bortezomib and could therefore reliably report on PSR activity (Fig. 2 B).

To determine how the UPS adapts to each stressor, we compared the relationship between UPS performance and PSR activation (Fig. 2 C). Perfect adaptation, a regime where cells respond to a stressor without any loss of UPS performance, would be observed as activation of the PSR without any change in UPS performance. “Nonadaptation” would manifest as a lack of PSR activation with concurrent loss of UPS performance. Partial adaptation would present as an intermediate between these two regimes, where the PSR activates but UPS performance still declines. Finally, overadaptation would be evidenced by PSR activation coupled to an increase in UPS performance. Strikingly, cells exhibited near-perfect adaptation in response to low doses of bortezomib (2.5 µM), as the PSR was activated but stability remained the same or nominally increased for both degrons (Fig. 2, A and D). At higher doses (>2.5 µM), the response to bortezomib resulted in partial adaptation, showing decreasing UPS performance as bortezomib dose increased despite PSR activation. Responses to canavanine and AZC caused a divergent response: UPS adaptation was perfect or overadaptive up to 200 µM and 1 mM, respectively, for Cyto-Deg but partial for ERm-Deg. These observations suggest UPS adaptation is highly effective but becomes less so under severe stressors, as noted by an increase in stability of both degrons at high doses of bortezomib and ERm-Deg at several doses of canavanine and AZC.

### Activating the PSR improves UPS performance under stress

**Figure 3.**
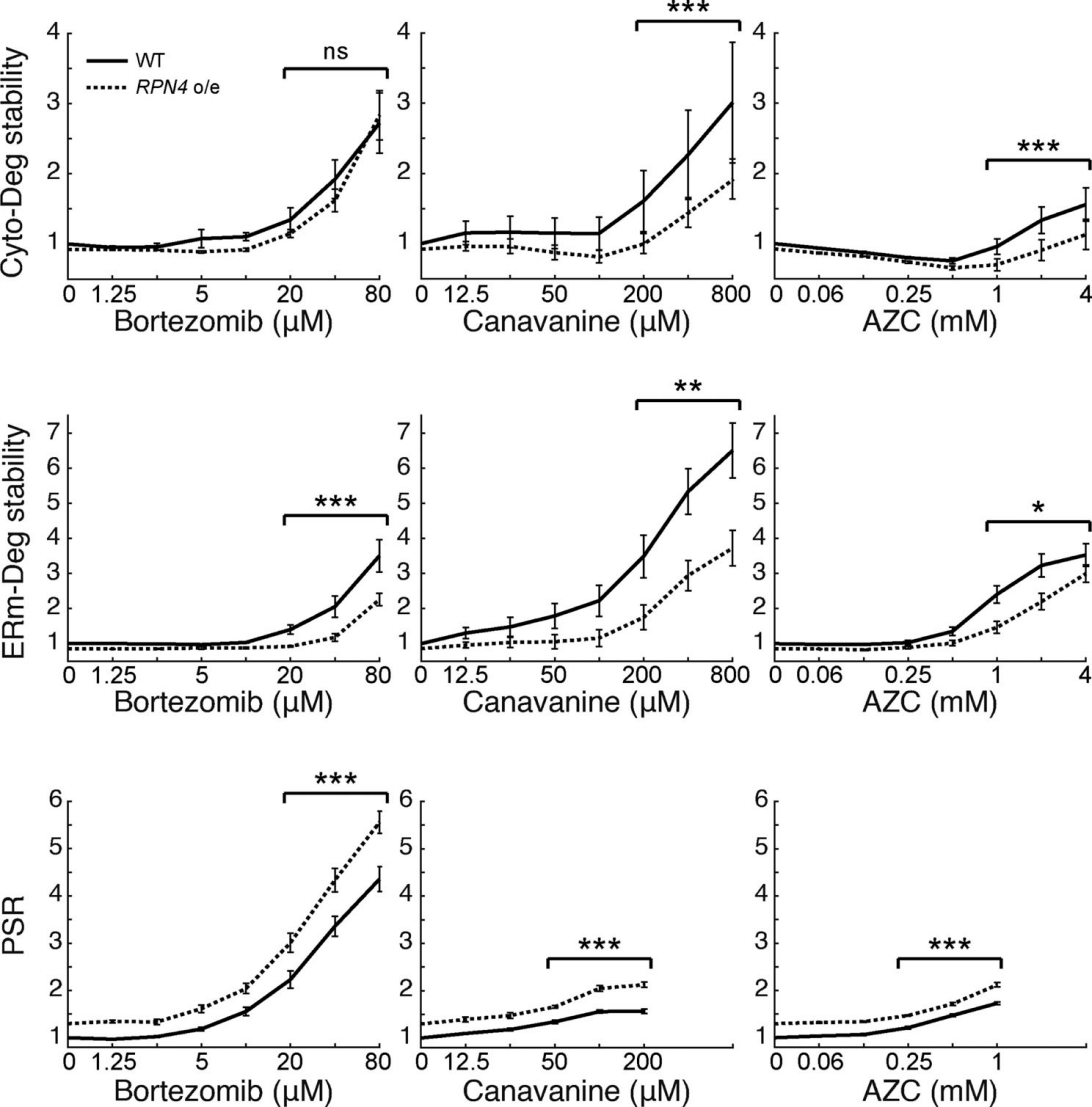
**Boosting the PSR improves UPS performance under stress.** Measurements of fold degron stability (top: Cyto-Deg; middle: ERm-Deg) and fold PSR activity (bottom) in titrations of bortezomib, canavanine, or AZC for cells expressing an empty vector (black, "WT") or a second copy of *RPN4*, causing overexpression (dotted, "*RPN4* o/e"). Significance was collectively tested across the three highest concentrations measured by combining data from these concentrations into one group per genetic context, then performing a paired two-sided Student’s *t* test. Error bars denote standard error for *n* ≥ 3 biological replicates. ns, P > 0.05; *, P < 0.05; **, P < 0.01; and ***, P < 0.001.

To understand the limitations of the PSR, we next investigated why adaptation was imperfect for ERm-Deg under AZC and canavanine treatment and for both degrons at high doses of bortezomib. One model to explain these results is that the PSR is insufficiently activated in these conditions, resulting in a PSR that cannot fully compensate for the increased proteotoxic burden. Alternatively, the stressors we applied may exceed the capacity of the PSR to increase UPS performance. To distinguish between these models, we tested whether enhancing the PSR by expressing a second copy of *RPN4* improves degradation in stress conditions. Indeed, it was recently shown that *RPN4* overexpression improves UPS performance in clearing mislocalized ER proteins or defective ribosome proteins in the cytosol (Schmidt et al., 2019; Tye et al., 2019). As expected, expressing a second copy of *RPN4* in the absence of stress increased the PSR and destabilized Cyto-Deg and ERm-Deg relative to an empty vector control (Fig. 1 E and Fig. 3). Under stress conditions, the addition of a second *RPN4* copy lowered degron stability for nearly all concentrations of bortezomib, canavanine, or AZC tested (Fig. 3). We conclude that activating the PSR is sufficient to improve UPS performance under all stressors and that Rpn4 is insufficiently activated to clear specific substrates in partially adaptive regimes.

### Folding stressors activate the PSR predominantly via transcription of *RPN4*

**Figure 4.**
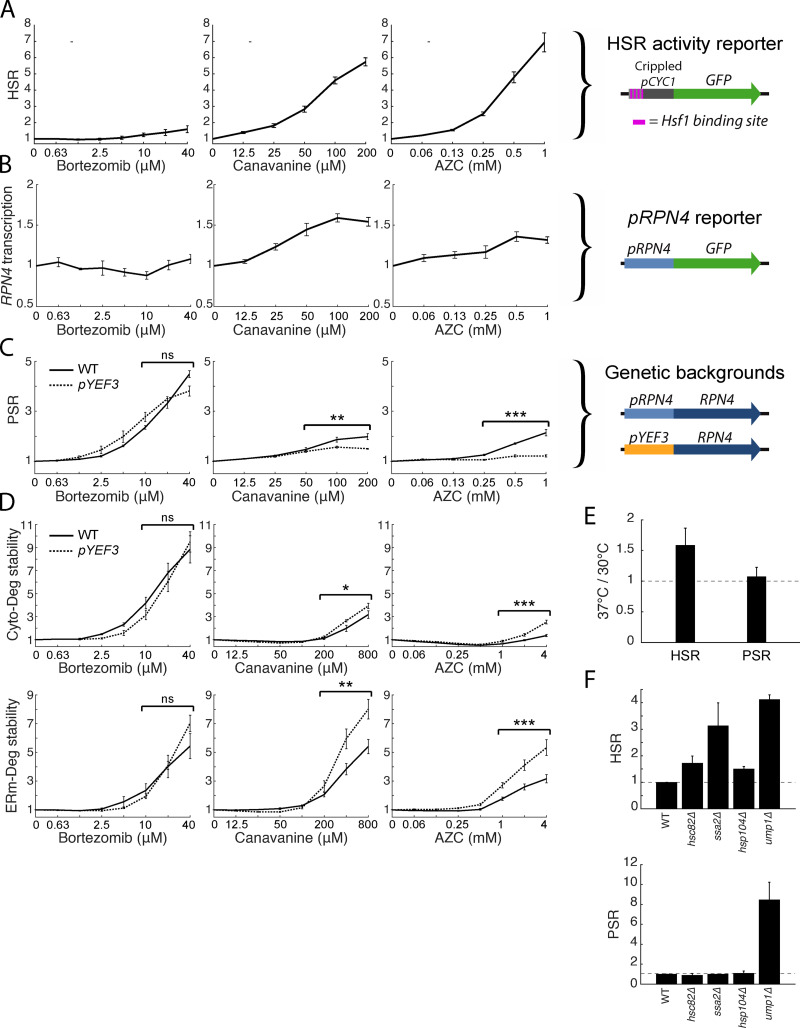
**Folding stressors activate the PSR via transcription of *RPN4* and do not increase UPS substrate load.** **(A)** Schematic of a HSR reporter (right) and its fold GFP induction in titrations of bortezomib, canavanine, and AZC (left). **(B)** Schematic of a *pRPN4* reporter (right) and its fold GFP induction in titrations of bortezomib, canavanine, and AZC (left). **(C)** Schematic of the *RPN4* locus in wild type or a *pYEF3:RPN4* background (right), and measurements of fold PSR activity in stressor titrations for both strains (left). **(D)** Measurements of Cyto-Deg (top) and ERm-Deg (bottom) fold stability for titrations of bortezomib, canavanine, or AZC in either a wild type (solid) or *pYEF3:RPN4* (dotted) background. Titrations of both strains are normalized to the no-treatment values of each reporter. **(E)** Mean fold activity of the HSR or PSR at 37°C relative to 30°C in wild-type cells. **(F)** Mean fold activity of the HSR (left) or PSR (right) relative to wild type upon deletion of *HSC82*, *SSA2*, *HSP104*, or *UMP1*. For titrations, significance was collectively tested across the three highest concentrations measured by combining data from these concentrations into one group per genetic context, then performing a paired two-sided Student’s *t* test. Error bars denote standard error for *n* ≥ 3 biological replicates. ns, P > 0.05; *, P < 0.05; **, P < 0.01; and ***, P < 0.001.

**Figure S1.**
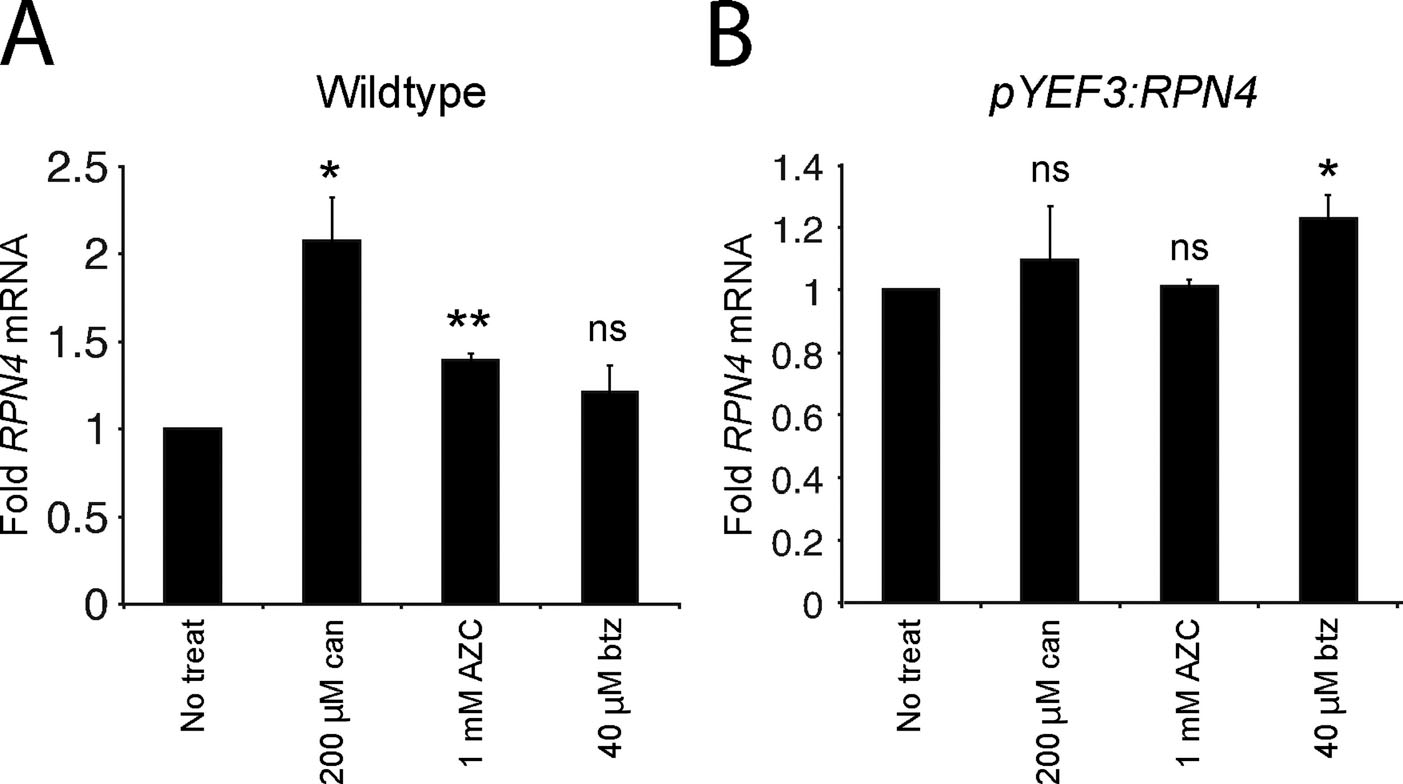
**Folding stressors increase *RPN4* mRNA in wild type but not in the *pYEF3:RPN4* background.** **(A and B)** Mean fold *RPN4* mRNA measured by RT-qPCR in wild-type yeast (A) or *pYEF3:RPN4* yeast (B) after treatment with canavanine, AZC, or bortezomib. Values are normalized to *ACT1* mRNA, then normalized to no treatment. Significance between the untreated and treated samples was determined by a paired two-sided Student’s *t* test. Error bars denote standard error for *n* = 3 biological replicates. ns, P > 0.05; *, P < 0.05; and **, P < 0.01. btz, bortezomib; can, canavanine; treat, treatment.

Because canavanine and AZC had qualitatively similar effects on each degron that were distinct from bortezomib (Fig. 2 D), we reasoned that folding and proteolytic stressors may be sensed differently by cells. Proteasome inhibition via bortezomib is likely to activate the PSR by impaired degradation of Rpn4, although it may also cause transcriptional activation of *RPN4*. In contrast, it is unclear whether the folding stressors canavanine and AZC activate the PSR through creation of new proteasome substrates that compete with Rpn4 for degradation and stabilize it (i.e., an indirect proteolytic stress), transcriptional activation of *RPN4*, or both. Indeed, canavanine and AZC robustly activate Hsf1, a transcriptional activator of *RPN4* (Hahn et al., 2006; Yamamoto et al., 2008; Alford and Brandman, 2018), and may activate other upstream transcription factors like Yap1 and Pdr1/3 as well. We therefore investigated the role of transcriptional regulation of *RPN4* in activating the PSR during stressor treatment. Consistent with previous work, a reporter of Hsf1 activity (Brandman et al., 2012) was robustly activated in the presence of canavanine and AZC (up to six- and sevenfold activation at their highest concentrations, respectively), with comparatively weak (up to twofold) maximal activation by bortezomib (Fig. 4 A). Because Hsf1 targets the *RPN4* promoter (Hahn et al., 2006), we predicted that the promoter of RPN4 (*pRPN4)* should be up-regulated in canavanine and AZC stress. We measured the activity of *pRPN4* using a *pRPN4:GFP* plasmid reporter and found that bortezomib did not activate *pRPN4*, while canavanine and AZC modestly increased the promoter’s activity (1.6- and 1.3-fold; Fig. 4 B). RT quantitative PCR (RT-qPCR) of *RPN4* mRNA in these conditions gave results in agreement with the fluorescent reporter (Fig. S1 A). These results are consistent with a model in which folding stressors but not proteolytic stressors up-regulate the PSR via transcriptional regulation of *RPN4*.

### Folding stressors do not induce proteolytic stress

Given the similarity in magnitude of PSR activation and *pRPN4* induction in canavanine and AZC (Fig. 2 A and Fig. 4 B), we hypothesized that PSR activation by these two stressors is fully accounted for by transcriptional targeting of *pRPN4*, with little or no contribution through stabilization of Rpn4. By contrast, bortezomib does not activate *pRPN4* (Fig. 4 B) and presumably induces the PSR through stabilization of Rpn4 alone. To determine the contribution of Rpn4 stabilization to the PSR under canavanine and AZC treatments, we disabled transcriptional regulation by engineering strains in which the genomic copy of *RPN4* is under the control of the *YEF3* promoter (*pYEF3*), which is not targeted by Hsf1 and exhibits the same approximate basal expression as *pRPN4* (1.23-fold basal; standard error = .027). In this background, PSR induction was retained in bortezomib treatment, but lost in canavanine and AZC treatment (Fig. 4 C). RT-qPCR verified that *RPN4* transcription levels remained constant under folding stress in the *pYEF3:RPN4* background (Fig. S1 B). We predicted that loss of promoter-mediated PSR activation would impair adaptation to folding stressors and minimally affect proteolytic stressors. Indeed, Cyto-Deg and ERm-Deg stability was sensitized to canavanine and AZC in the *pYEF3:RPN4* background, evidenced by greater degron stabilization relative to wild type (Fig. 4 D). Furthermore, degron stabilization was greater under AZC treatment than canavanine treatment, which correspondingly had a greater loss of PSR activation in the *pYEF3:RPN4* background. We conclude that protein folding stress caused by canavanine and AZC increases *RPN4* transcription with little or no effect on Rpn4 stability.

To test if folding stressors generally do not change Rpn4 stability, we employed additional approaches to cause folding stress and measured their effects on the PSR. As a general folding stressor, we increased the likelihood of protein misfolding by raising the steady-state temperature of wild-type yeast from 30°C to 37°C. This was sufficient to activate the HSR, but caused no change in PSR activation (Fig. 4 E). We next deleted several protein chaperone genes (*HSC82*, *SSA2*, and *HSP104*) implicated in proteome integrity (Borkovich et al., 1989; Craig and Jacobsen, 1984; Sanchez and Lindquist, 1990) and one proteasome assembly chaperone (*UMP1*) implicated in proper proteasome function (Ramos et al., 1998) and measured their effect on the HSR and PSR. All deletions increased the HSR, but only deletion of *UMP1* (a direct effector of the proteasome) activated the PSR (Fig. 4 F). We conclude that endogenous proteins that misfold due to folding stressors do not induce proteolytic stress.

### Folding stressors induce protein aggregation, but proteolytic stressors do not

**Figure S2.**
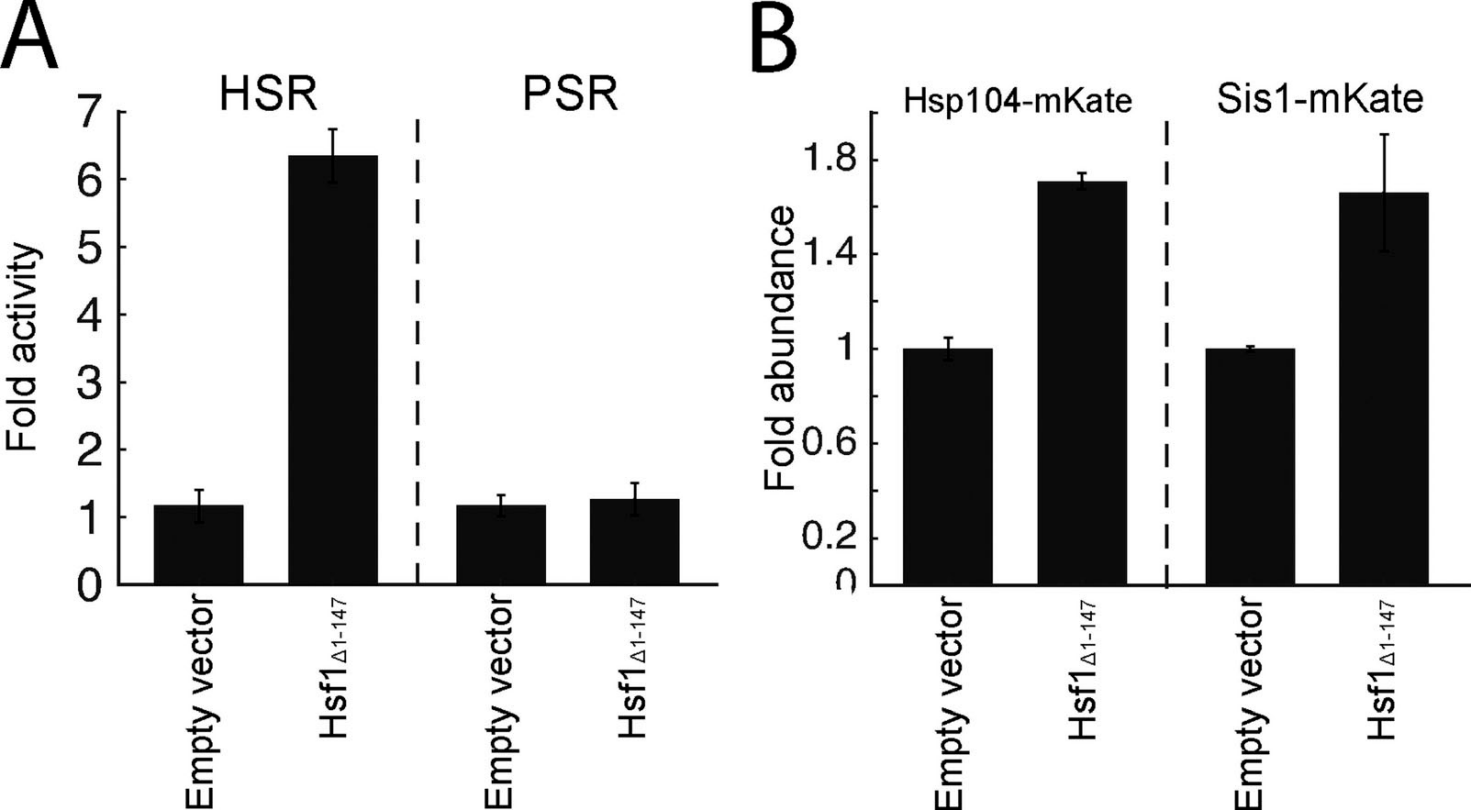
**Expression of Hsf1_Δ1-147_ activates the HSR and its canonical targets, Hsp104 and Sis1.** **(A) **Mean fold activity of the HSR or PSR in cells expressing either an empty plasmid vector or a vector expressing *HSF1_Δ1-147_*. **(B)** Mean fold change in levels of Hsp104-mKate or Sis-mKate in cells expressing either an empty plasmid vector or a vector expressing *HSF1_Δ1-147_*. Error bars denote standard error for *n* = 3 biological replicates.

**Figure 5.**
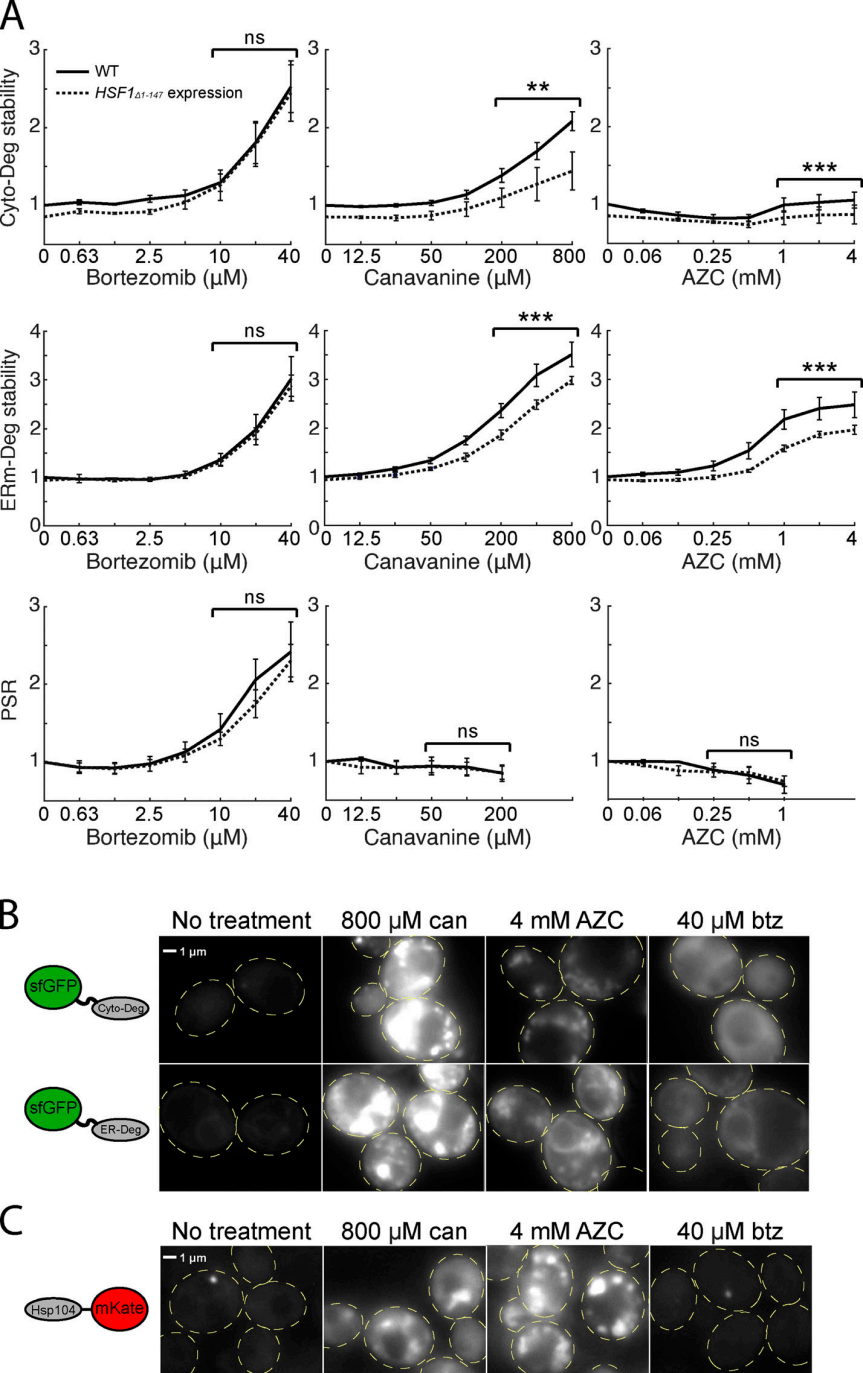
**Folding stressors cause aggregation and result in failure to target aggregation-prone substrates to the proteasome. (A)** Measurements of fold degron stability (top: Cyto-Deg, middle: ERm-Deg) and fold PSR activity (bottom) in titrations of bortezomib, canavanine, or AZC for cells expressing an empty vector (solid) or a copy of *HSF1_Δ1-147_* (dotted). Significance was collectively tested across the three highest concentrations measured by combining data from these concentrations into one group per genetic context, then performing a paired two-sided Student’s *t* test. Error bars denote standard error for *n* ≥ 3 biological replicates. **(B and C)** Fluorescent localization of ERm-Deg (GFP), Cyto-Deg (GFP), or Hsp104-mKate2 (RFP) in cells treated with 800 µM canavanine, 4 mM AZC, 40 µM bortezomib, or no treatment for 5 h. Cells are outlined in yellow dashed lines. ns, P > 0.05; **, P < 0.01; and ***, P < 0.001. btz, bortezomib; can, canavanine.

Given that the levels of misfolded proteins generated by folding stressors increased yet the proteasome burden appeared not to increase, we explored the possibility that the misfolded proteins are instead sequestered into aggregates in which they are protected from proteasomal degradation. Indeed, it has been previously reported that AZC can cause aggregation of endogenous proteins (Weids and Grant, 2014), and that aggregation can be a mechanism for avoiding degradation (Wallace et al., 2015). Because canavanine and AZC robustly activate the HSR, a response that is driven by a drop in protein chaperone availability (Zheng et al., 2016; Alford and Brandman, 2018), we reasoned that chaperones become limiting under folding stressors, and this may drive protein aggregation. To test this, we boosted Hsf1 activity to increase chaperone levels and determined if this would increase the stability of our degron reporters during canavanine and AZC treatment. We expressed an extra copy of Hsf1 with an N-terminal truncation that renders it constitutively active (Hsf1_Δ1-147_; Sorger, 1990) in a strain expressing *RPN4* from the *CYC1* promoter (to eliminate the confounding effect that Hsf1 activates *pRPN4*). Indeed, expression of Hsf1_Δ1-147_ activated the HSR and increased the levels of Hsp104 and Sis1, two canonical targets of the HSR (Fig. S2; Amorós and Estruch, 2001; Solís et al., 2018). PSR activity was unchanged upon expression of Hsf1_Δ1-147_, suggesting either that Hsf1_Δ1-147_ poorly targets the *RPN4* promoter, or that there exists a compensatory mechanism reducing PSR activity. Hsf1_Δ1-147_ reduced Cyto-Deg and ERm-Deg levels in response to canavanine and AZC but not bortezomib (Fig. 5 A). These results suggest that the decrease in UPS performance due to canavanine and AZC is resolvable by increasing chaperone levels, supporting that they cause protein folding stress, while bortezomib does not. This chaperone dependence for degradation is consistent with the hypothesis that folding stressors result in sequestration of UPS substrates into aggregates.

**Figure S3.**
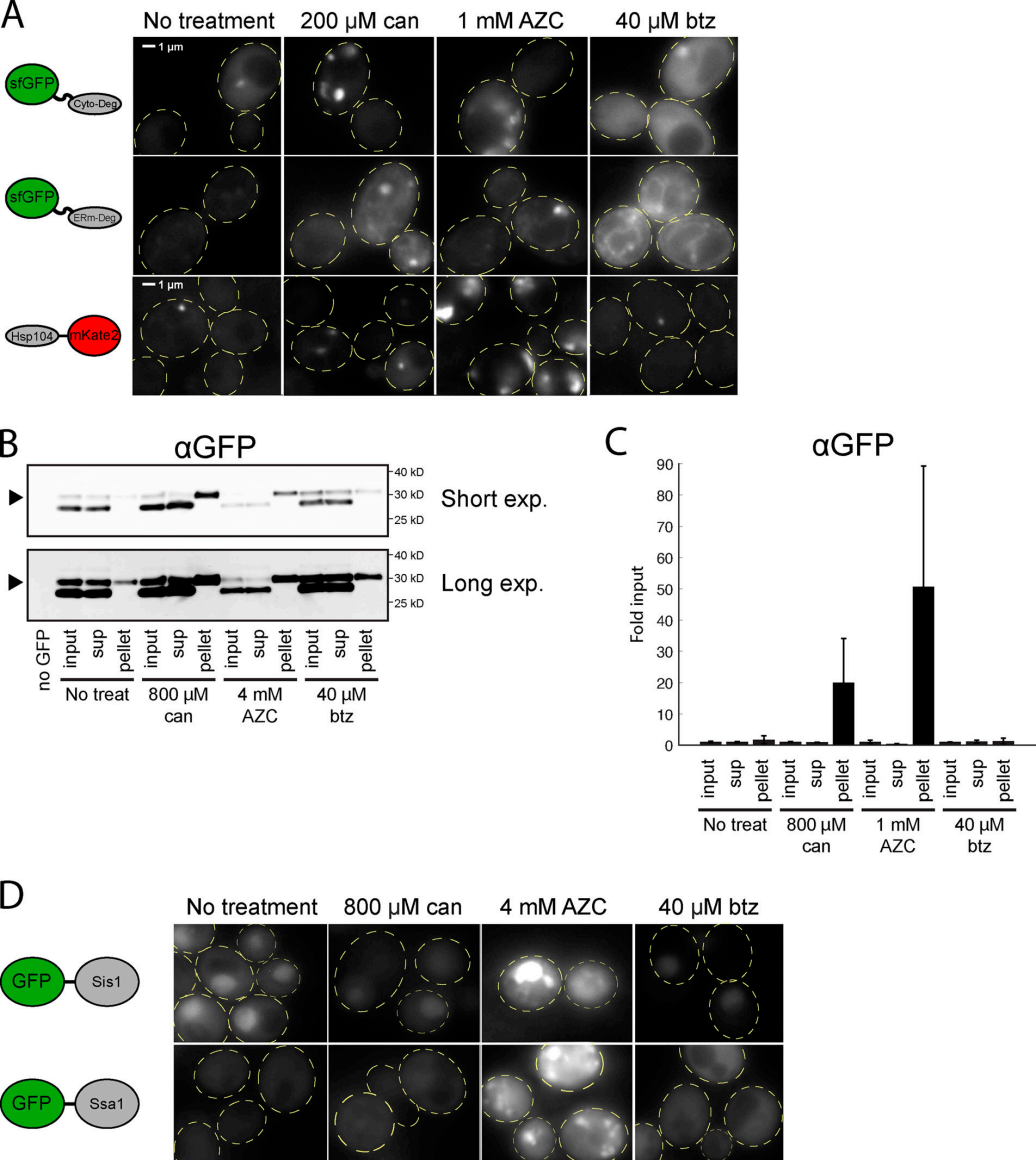
**Folding stressors cause aggregation and recruitment of heat shock proteins.** **(A) **Fluorescent localization of ERm-Deg (GFP), Cyto-Deg (GFP), or Hsp104-mKate2 (RFP) in cells treated with 200 µM canavanine, 1 mM AZC, 40 µM bortezomib, or no treatment for 5 h. Cells are outlined in yellow dashed lines. **(B)** Immunoblot of Cyto-Deg after cells were treated with canavanine, AZC, or bortezomib. Aggregated material was collected from the cells by centrifugation, and the input, supernatant (sup), and pellet were immunoblotted. Arrows denote the position of Cyto-Deg. **(C)** Quantification of *n* = 3 biological replicates of the immunoblot in B. Values are normalized to the respective input of each sample. **(D) **Fluorescent localization of GFP-Sis1 and GFP-Ssa1 in cells treated with 800 µM canavanine, 4 mM AZC, 40 µM bortezomib, or no treatment for 5 h. btz, bortezomib; can, canavanine; Exp., exposure; treat, treatment.

**Figure S4.**
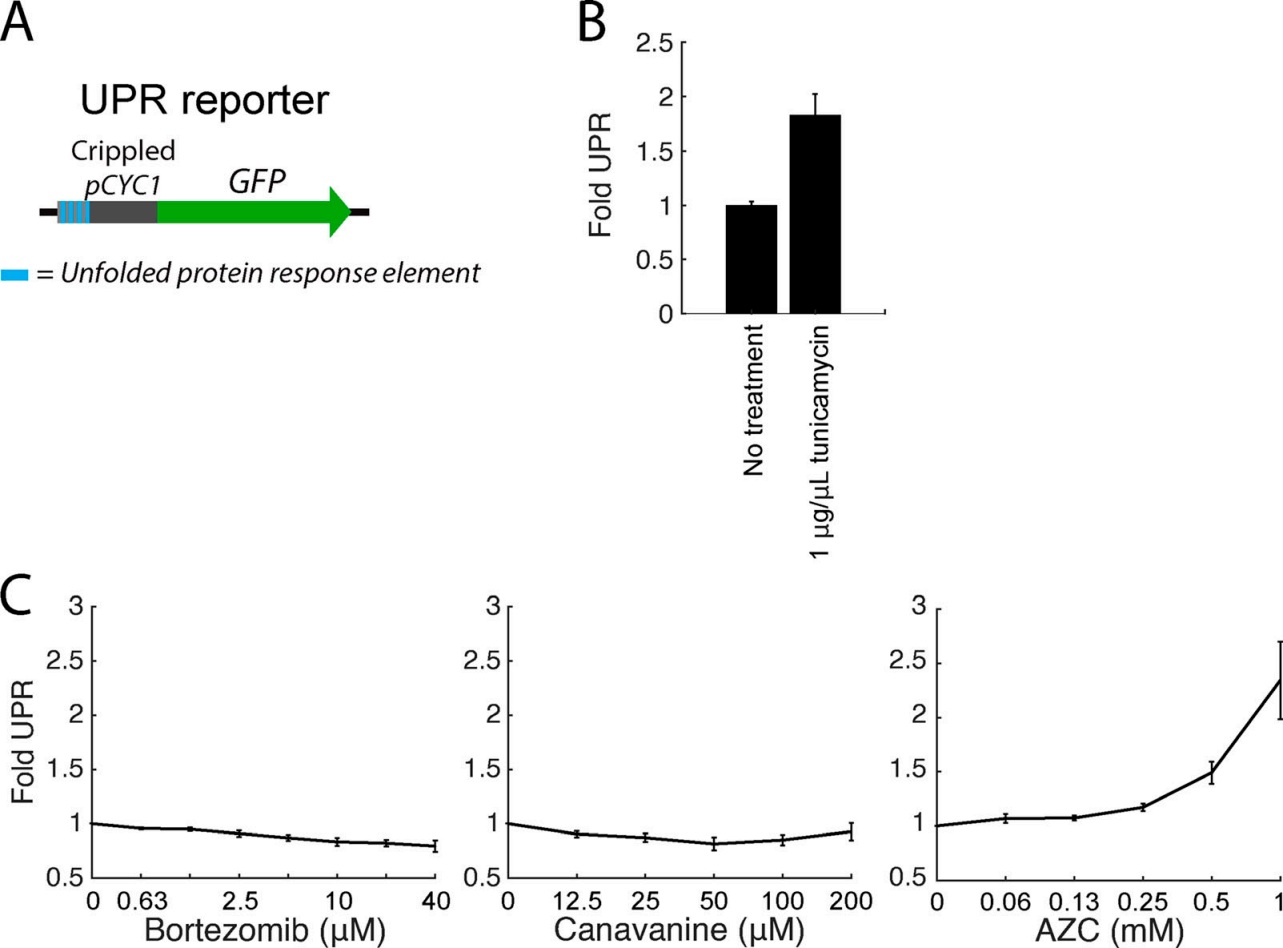
**Unfolded protein response (UPR) activation under folding and proteolytic stressors.** **(A)** Schematic of the UPR reporter. **(B)** Mean fold UPR activity upon treatment with 1 µg/µl tunicamycin for 5 h. **(C)** Mean fold UPR activity upon treatment with a titration of botezomib, canavanine, or AZC. Error bars denote standard error for *n* = 3 biological replicates.

To directly assess the presence of aggregates in canavanine- and AZC-treated cells, we performed fluorescence microscopy on cells expressing Cyto-Deg or ERm-Deg. Canavanine and AZC caused GFP in both Cyto-Deg and ERm-Deg to form inclusions (Fig. 5 B and Fig. S3 A). In support of these observations, we biochemically isolated aggregated material from cells expressing Cyto-Deg and found that Cyto-Deg was greatly enriched in the aggregated fraction upon treatment with canavanine or AZC but not bortezomib (Fig. S3, B and C). AZC and canavanine increased expression of Hsp104 and relocalized it into foci in cells without a degron reporter (Fig. 5 C). Furthermore, the chaperones Sis1 and Ssa1 localized into inclusions upon AZC treatment (Fig. S3 D). This change in chaperone localization suggests that a population of endogenous proteins (not just our synthetic reporters) is sequestered into aggregates in the presence of canavanine and AZC. Indeed, the binding of Ssa1 to client proteins is associated with Hsf1 activation (Krakowiak et al., 2018), a phenomenon observed in folding stress conditions (Fig. 4 A). By contrast, even a high dose (40 µM) of bortezomib that strongly increased degron levels did not alter the localization of the degrons or induce Hsp104 expression. This paradigm likely also applies to the ER, as AZC but not bortezomib led to an increase in unfolded protein response signaling (Fig. S4). These observations suggest that canavanine and AZC cause protein folding stress that drives misfolded proteins into aggregates rather than targeting them to the proteasome and that proteasomal stressors do not induce protein aggregation. Under this model, folding stressors fail to stabilize Rpn4 because they do not increase the total amount of proteasome substrates that compete with Rpn4 for degradation.

**Figure S5.**
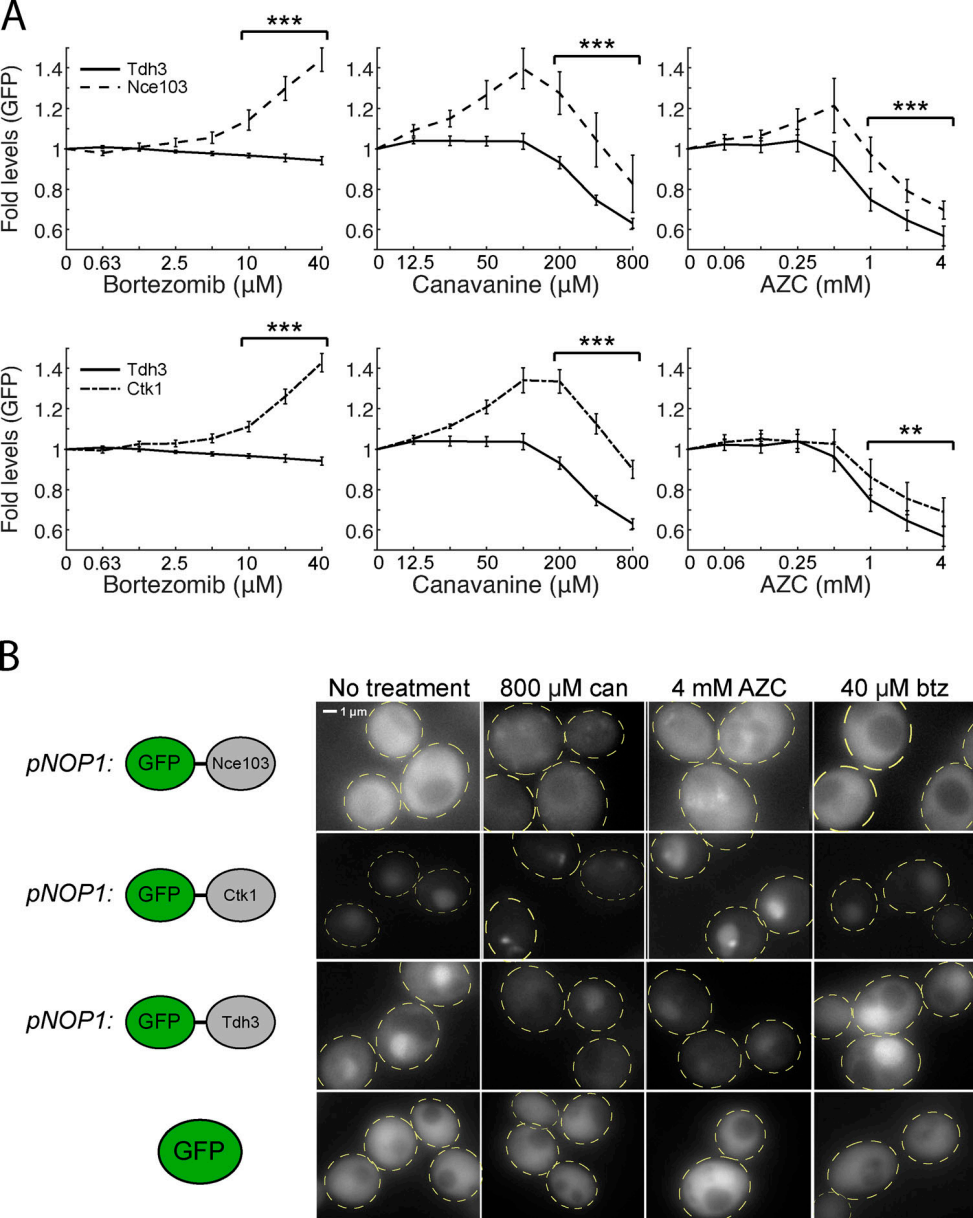
**Short-lived proteins Nce103 and Ctk1 under folding and proteolytic stressors.** **(A)** Measurements of fold protein (top: GFP-Nce103 and GFP-Tdh3; bottom: GFP-Ctk1 and GFP-Tdh3) levels in titrations of bortezomib, canavanine, or AZC. Significance was collectively tested across the three highest concentrations measured by combining data from these concentrations into one group per genetic context, then performing a paired two-sided Student’s *t* test. Error bars denote standard error for *n* > 3 biological replicates. **(B)** Fluorescent localization of GFP-Nce103, GFP-Ctk1, GFP-Tdh3, and free GFP in cells treated with 800 µM canavanine, 4 mM AZC, 40 µM bortezomib, or no treatment for 5 h. Free GFP is expressed from *pTDH3*. Cells are outlined in yellow dashed lines. **, P < 0.01; and ***, P < 0.001. btz, bortezomib; can, canavanine.

While Cyto-Deg and ERm-Deg serve as quantitative reporters for UPS performance, we wished to determine whether their behaviors are also shared by endogenous proteins. To achieve this, we identified endogenous proteins that are targeted for degradation by examining proteins with short half-lives (Christiano et al., 2014). We selected Nce103 and Ctk1, two short-lived proteins (t_1/2_ < 30 min), and measured levels of N terminally GFP tagged versions of these proteins expressed from the constitutive high-expression *NOP1* promoter (Weill et al., 2018). Both of these proteins increased in abundance upon treatment with bortezomib, canavanine, or AZC relative to the long-lived control protein Tdh3 (*t*_1/2_ > 12 h; Christiano et al., 2014; Fig. S5 A). Furthermore, Nce103 and Ctk1 formed inclusions upon treatment with canavanine or AZC, but remained diffuse upon treatment with bortezomib (Fig. S5 B). Tdh3 and free GFP remained diffuse in all treatments. Combining these observations with our observations of protein chaperones that bind endogenous proteins (Fig. 5 C and Fig. S3 D), we conclude that localization into inclusions during folding stress and a lack of inclusion formation during proteolytic stress are general behaviors of endogenous proteins under proteotoxic stressors.

**Figure 6.**
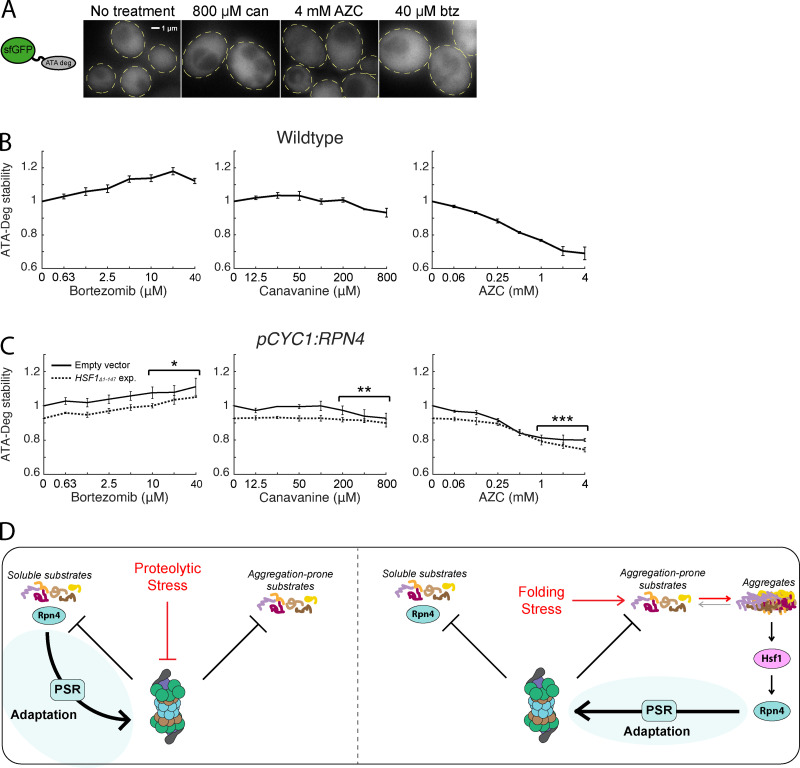
**Folding stressors do not impair degradation of soluble UPS substrates.** **(A)** Fluorescent localization of ATA-Deg (GFP) in cells treated with 800 µM canavanine, 4 mM AZC, 40 µM bortezomib, or no treatment for 5 h. Cells are outlined in yellow dashed lines. **(B)** Measurements of fold ATA-Deg stability in titrations of bortezomib, canavanine, or AZC. **(C)** Fold ATA-Deg stability in drug titrations in cells expressing an empty vector (solid) or a copy of *HSF1_Δ1-147_* (dotted). Significance was collectively tested across the three highest concentrations measured by combining data from these concentrations into one group per genetic context, then performing a paired two-sided Student’s *t* test. Error bars throughout denote standard error for *n* ≥ 3 biological replicates. **(D)** Model for UPS adaptation by the PSR to proteolytic and folding stressors. Left: Proteolytic stressors increase the stability of all proteasome substrates. This includes Rpn4, whose accumulation leads to PSR activation. Right: Folding stressors causes some proteasome substrates to sequester into aggregates. Aggregation triggers the HSR, activates transcription of *RPN4*, and causes PSR activation. *, P < 0.05; **, P < 0.01; and ***, P < 0.001. btz, bortezomib; can, canavanine; exp., exposure.

If aggregation interferes with the degradation of misfolded proteins by the UPS, we predicted that highly soluble proteasome substrates that escape aggregation during folding stress would continue to be degraded normally. To test this prediction, we built a third degron reporter consisting of sfGFP fused to the six-peptide sequence "ATAATA" (ATA-Deg), which was demonstrated in previous work to be poly-ubiquitylated and targeted for proteasomal degradation, but whose degron sequence is soluble (Sitron and Brandman, 2019). Accordingly, ATA-Deg was diffuse throughout the cytosol even under high doses of canavanine, AZC, or bortezomib (Fig. 6 A). Bortezomib treatment increased the stability of ATA-Deg, indicating sensitivity to proteolytic stress. Conversely, ATA-Deg levels were constant or decreased under all concentrations of canavanine and AZC treatment (Fig. 6 B), suggesting that folding stress does not impair degradation of ATA-Deg. Consistent with this and in contrast to Cyto-Deg and ERm-Deg (Fig. 5 A), ATA-Deg stability was not affected by expression of Hsf1_Δ1-147_ under folding stressors (Fig. 6 C). We conclude that folding stressors sequester aggregation-prone UPS substrates without disrupting degradation of soluble substrates (Fig. 6 D).

## Discussion

Here we assessed the performance and adaptability of the UPS in yeast under stress conditions using quantitative measurements of UPS performance and the adaptive transcriptional response of the UPS (PSR). We found that proteolytic and protein folding stressors stabilized misfolded proteins through separate, nonoverlapping mechanisms, with the former blocking degradation of misfolded proteins and the latter generally resulting in their aggregation rather than their targeting to the proteasome. Despite a difference in the underlying proteostasis defect, the UPS productively responded to both proteolytic and folding stressors, and in both cases, this included perfect or near-perfect adaptation (no loss in degradation performance) for some substrates (Fig. 2 D).

The perfect and near-perfect adaptation we observed for the UPS implies the existence of an underlying network that can mechanistically achieve this (Ferrell, 2016). In the case of folding stress, *RPN4* is activated transcriptionally (Fig. 4, B and C), likely by Hsf1 and possibly by other factors like Yap1 and Pdr1/3, to achieve perfect adaptation for proteins that aggregate in the cytosol. This intervention may cause increased degradation rates of soluble proteasome substrates, an intriguing consequence of a system that tunes the UPS to “problem” proteins that are poor UPS substrates (aggregated proteins). This substrate-specific adaptation likely occurs to some degree in all stress responses that use concerted transcriptional regulation to address substrates with distinct adaptive needs. Future studies into additional forms of proteotoxic stress and those that monitor proteasome regulation without requiring transcription and protein synthesis (as does our PSR reporter) will further illuminate the extent and nature of PSR activity under stress.

Under proteolytic stress, where Rpn4 is stabilized and its activation rescues the degradation of other proteasomal substrates (Fig. 2 D), perfect adaptation requires that the increase in Rpn4 activity compensate for the loss in degradation of proteasome substrates. This may occur via a combination of the multiple degrons present on Rpn4 and could also involve post-translational regulation of Rpn4, which is ubiquitylated and phosphorylated (Ju et al., 2007; Wang et al., 2004). Additionally, proteasome inhibition may cause the buildup of substrates that selectively outcompete Rpn4 but not other substrates, making Rpn4 levels hypersensitive to proteolytic stress. Understanding the range of substrates and conditions for which perfect or near-perfect adaptation occurs and how different substrates may be prioritized for degradation relative to Rpn4 is an important topic for future study.

Our data suggest that protein folding stressors do not burden the proteasome with increased overall substrate load (Fig. 4, C, F, and G). Instead, misfolding causes the sequestration of aggregation-prone substrates into inclusions and a resultant loss of their UPS targeting (Fig. 5, B and C). This cellular behavior that favors aggregation over degradation is contrary to what has been observed for certain thermolabile proteins, which presumably misfold upon temperature increase and are degraded (Betting and Seufert, 1996; Dohmen et al., 1994; Downey et al., 2006). Our work instead suggests that endogenous proteins in yeast have evolved to aggregate rather than become targeted by the UPS during folding stress. This conclusion is in line with experiments demonstrating that the yeast proteome forms aggregates instead of being targeted to the UPS in response to acute heat shock (Wallace et al., 2015). Such a strategy has the advantage of preserving proteins that may be refolded at a later time after stressors are removed, allowing a faster recovery with less energy expenditure. It may also prevent adverse effects of high UPS activity, which has been shown to confer growth defects (Wang et al., 2010). However, by promoting aggregation, cells create a risk for proteotoxicity and entrance into a pathogenic state, as observed in the numerous neurodegenerative diseases characterized by buildup of misfolded proteins in neurons (Labbadia and Morimoto, 2015; Sweeney et al., 2017). Even inert insoluble proteins can cause growth defects (Geiler-Samerotte et al., 2011). While we do not rule out the possibility that some endogenous proteins are targeted to the proteasome upon misfolding, our data suggest that the frequency of such events is insufficient to increase the overall burden on the UPS during folding stress. Moving forward, it remains to be determined whether human cells, particularly neurons, make a similar tradeoff that favors aggregation over degradation and whether this tradeoff puts them at risk for disease. Future studies evaluating the fitness cost of perturbing the PSR and other systems will help clarify the evolutionary basis for these systems.

Because there is no surge of proteasome substrates when cells are faced with folding stressors, up-regulation of the PSR in these conditions relies entirely upon the stress-sensitive transcriptional up-regulation of *RPN4* rather than stabilization of the Rpn4 protein. Cells may have a divergent ability to sense different protein quality failures, as suggested by better adaptation of a cytosolically localized degron (Cyto-Deg) vs. an ER membrane–localized one (ERm-Deg) under folding stressors. Despite the absence of a UPS “traffic jam,” up-regulation of the PSR during these stressors still improves the cell’s ability to degrade aggregation-prone proteins (Cyto-Deg and ERm-Deg in AZC and canavanine treatment). This could be because of enhanced PSR activity coinciding with the emergence of aggregates limits their formation, or because misfolded proteins are in an equilibrium between aggregated and soluble states, and boosting the PSR adjusts this equilibrium point. Exploration of these possibilities is an exciting prospect for preventing and reversing diseases characterized by protein aggregation.

## Materials and methods

### Yeast strain construction and culturing methods

All yeast strains were created from BY4741. Yeast cultures were grown at 30°C (unless otherwise noted) in yeast extract peptone dextrose media or synthetic dropout (SD) media.

Strain construction was done by transforming cells with a crude PCR product bearing 40 base pair overhangs homologous to the target genomic locus, a selection cassette for either antibiotic selection in yeast extract peptone dextrose or auxotrophic selection in SD, and any other desired sequences. Deletion strains were constructed in the BY4741 background via transformation with antibiotic selection cassettes (NATMX6 or KANMX6), amplified with overhangs flanking the open reading frame to be deleted. The *pCYC1:RPN4* and *pYEF3:RPN4* strains were constructed via transformation by inserting a His3 cassette and 1000nt of either *pCYC1* or *pYEF3* immediately upstream of the *RPN4* open reading frame. The Hsp104-mKate2 and Sis1-mKate2 strains were generated via transformation by inserting the yeast-optimized mKate2 coding sequence and a hygromycin selection cassette immediately downstream of the endogenous *HSP104* or *SIS1* open reading frame. The free GFP strain was generated via transformation by inserting the yeast-optimized sfGFP coding sequence and a *URA3* selection cassette into the *ura3Δ* locus. Several GFP-tagged protein strains (Nce103, Ctk1, Tdh3, Sis1, and Ssa1) were a gift from U. Weill and M. Schuldiner (Weizmann Institute of Sciences, Rehovot, Israel; Weill et al., 2018). All transformants were verified by genomic PCR.

Plasmids used in this study were cloned using the Gibson Assembly method and the NEBuilder HiFi DNA Assembly Master Mix (New England Biolabs). The PSR reporter, HSR reporter, *pRPN4:GFP* reporter, Cyto-Deg, ER-Deg, and ATA-Deg plasmids were expressed from high-copy plasmids containing a Ura3 selection cassette. For *RPN4* overexpression experiments, a single-copy plasmid with a His5 selection cassette and either *pRPN4:RPN4* or no insert was coexpressed with the fluorescent reporters mentioned above. For experiments involving the expression of truncated *HSF1*, a single-copy plasmid with a Leu2 selection cassette and either *pHSF1:HSF1_Δ1-147_* or no insert was coexpressed with the fluorescent reporters mentioned above.

The degron sequence for Cyto-Deg is SIFYHIGTDLWTLSEHYYEGVLSLVASVIISGR, and the degron sequence for ERm-Deg is GVKHFVFFTMFSIMPAINFPLGR (“10-31” and “10-21” from Maurer et al., 2016). Both sequences were connected to sfGFP by a Gly-Ser-Gly-Ser linker.

### Fluorescent reporter assay measurements

All experiments were performed with at least three biological replicates measured on different days. For experiments testing the effects of drug stressors, stock solutions of the drugs were prepared in advance (5 mM bortezomib in ethanol, 0.5 M canavanine in water, and 0.5 M AZC in water). Yeast were inoculated into selective SD media such that after overnight growth (>12 h) in aerated culture tubes, their OD600 was between 0.05 and 0.3. Yeast were then diluted to 0.05 in a 96-well plate and incubated for 30 min. The drug stressors were serial diluted to 50x concentrations, then added to cells 1:50 to reach 1x concentrations. The yeast were grown for 5 h while shaking at 1,050 rpm. Fluorescence was measured on a BD Accuri C6 flow cytometer (BD Biosciences).

Measurements of cells treated with multiple drugs (Fig. 2 C) differed in that drug stressors were added at two distinct time points according to the schematic in Fig. 2 C. Measurements of the knockout strains (Fig. 4 F) differed in that upon overnight growth, cells with OD600 between 0.05 and 0.3 were immediately measured. Comparative measurements of cells in 30°C or 37°C growth (Fig. 4 E) differed in that they were grown overnight to saturation at 30°C, diluted to log phase, and grown for 5 h at 30°C, then split for growth at 30°C or 37°C at dilutions such that they were in log phase after overnight growth, then immediately measured.

### Reporter quantification

All quantitative analysis was performed using MATLAB v8.6 (MathWorks). For the PSR, HSR, and *pRPN4:GFP* reporters, the GFP fluorescence measurements were normalized to forward scatter for each cell. For ERm-Deg, Cyto-Deg, and ATA-Deg, GFP fluorescence measurements were normalized to RFP. In experiments comparing genetic backgrounds or growth temperatures (Fig. 1 E; and Fig. 4, E and F), samples were normalized to a corresponding wild-type control, which was set to 1. In titration experiments, samples were normalized to a corresponding no-treatment control that was set to 1. For titrations in backgrounds being compared with a control (the dashed lines in Fig. 3; Fig. 4, C and D; Fig. 5 A; and Fig. 6 C), the no-treatment control was set to its mean fold value relative to the no-treatment control in solid black.

### Imaging

Cells expressing Cyto-Deg, ERm-Deg, ATA-Deg, Hsp104-mKate2, GFP, or GFP-tagged proteins were inoculated into selective SD media such that after overnight growth (>12 h) in aerated culture tubes, their OD600 was between 0.1 and 0.4. Yeast were diluted to 0.1 and incubated for 30 min. Drug stressors were then added, and the cells were incubated for 5 h. Cells were concentrated through centrifugation and resuspension in SD media, then immobilized on glass slides pretreated with concanavalin A.

Imaging was performed at room temperature on an Eclipse 80i microscope (Nikon) with an X-Cite 120LED light source (Excelitas Technologies) and using a 100× 1.40 NA oil immersion lens, controlled via MetaMorph v7.10.2.240 software (Molecular Devices). Images were captured with an Andor DR-328G-CO1-SIL Clara CCD monochrome camera (Andor Technology). Hsp104 localization was determined by the detection of its mKate2 tag. All other species were detected by a superfolder GFP tag. The brightness and contrast of images were adjusted using MATLAB v8.6 (MathWorks).

### Immunoblotting

SDS-PAGE was performed on samples using Novex Nupage 4–12% Bis-Tris gels (Thermo Fisher Scientific). Samples were then transferred onto 0.45-µm nitrocellulose membranes (Bio-Rad) using a standard wet transfer protocol. Membranes were blocked with 5% milk in Tris-buffered saline with Tween for 1 h at room temperature. They were then stained with 1:2,000 Pierce monoclonal mouse anti-HA (26183; Thermo Fisher Scientific) primary antibody overnight at 4°C, followed by IRDye 800CW donkey anti-mouse (LiCor Biosciences) for 3 h at room temperature. Membranes were then scanned using a LiCor Odyssey (LiCor Biosciences).

### RT-qPCR

Yeast were inoculated into selective SD media such that after overnight growth (>12 h) in aerated culture tubes, their OD600 was between 0.1 and 0.5. They were then diluted to an OD600 of 0.1 and treated with 40 µM bortezomib, 200 µM canavanine, or 1 mM AZC for 5 h. They were then centrifuged at 7,000 *g* and flash-frozen with liquid nitrogen. RNA was extracted from the yeast via standard acid-phenol:chloroform extraction. 10 µg of RNA from each sample was DNase-treated (Turbo DNase; Thermo Fisher Scientific), cleaned and concentrated (AxyPrep Mag PCR Clean-Up; Axygen), and reverse-transcribed (Multiscribe RTase; Thermo Fisher Scientific) using random hexamer primers. The resulting cDNA samples were diluted twofold and combined with 2× SYBR green quantitative PCR mix (Luna; New England Biolabs) and 500 nM primers to measure the cycle threshold values of *RPN4* and *ACT1*, with *ACT1* serving as a loading control. The negative exponents of these values supplied relative mRNA concentrations that were then normalized to the no-treatment condition. The primers used for this procedure were as follows: *RPN4* forward, 5′-AGA​TGA​ACG​GGG​ACA​TGA​GA-3′; *RPN4* reverse, 5′-GAG​CTT​ACT​ACA​GCT​GAT​GTA​GG-3′; *ACT1* forward, 5′-CCG​GTG​ATG​GTG​TTA​CTC​AC-3′; *ACT1* reverse, 5′-ATG​GAA​GAT​GGA​GCC​AAA​GC-3′.

### Aggregated material collection

Aggregated material was collected in a manner similar to one that has been previously described (Rand and Grant, 2006). Specifically, yeast were inoculated into selective SD media such that after overnight growth (>12 h) in aerated culture tubes, their OD600 was between 0.1 and 0.5. They were then diluted to an OD600 of 0.1 and treated with 40 µM bortezomib, 800 µM canavanine, or 4 mM AZC for 5 h. Equivalent cell numbers (10 OD600 units) were centrifuged at 7,000 *g*, washed, and resuspended in 300 µl of lysis buffer (50 mM potassium phosphate buffer, pH 7, 1 mM EDTA, 5% [vol/vol] glycerol, 1 mM phenylmethylsulfonyl fluoride, and Pierce Protease Inhibitor [Thermo Fisher Scientific]). Cells were flash-frozen with liquid nitrogen, thawed, then incubated with 100 µl of lyticase (10 kU/ml; Sigma-Aldrich) for 30 min at 30°C. Cells were disrupted using a 1:3 volume of 0.5-mm glass beads (BioSpec Products, Inc.) and vortexing for 3 × 1 min. Each mixing was followed by 1 min of cooling on ice. Intact cells were removed by centrifugation at 3,000 *g* for 15 min. An input sample was collected (30 µl), and then aggregated proteins were collected by centrifugation at 15,000 *g* for 20 min. The supernatant was set aside, and then the pelleted samples were washed twice with a 4:1 mixture of lysis buffer and 10% Igepal CA-630 (NP-40; Sigma-Aldrich) and centrifuged at 15,000 *g* for 20 min. The samples were then resuspended in 60 µl of lysis buffer. The input, supernatant, and pellet samples were then immunoblotted using the method described above, but using 1:2,000 Invitrogen monoclonal mouse anti-GFP (MA5-15256; Thermo Fisher Scientific) as the primary antibody.

### Statistical significance

All statistical significance was tested by using a paired two-sided Student’s *t* test. For titration data, significance was collectively tested across the three highest concentrations measured by first combining data from these concentrations into one group per genetic context. Significance is denoted as ns (not significant), P > 0.05; *, P < 0.05; **, P < 0.01; and ***, P < 0.001. Data distribution was assumed to be normal, but this was not formally tested.

### Online supplemental materials

Fig. S1 shows that folding stressors increase *RPN4* mRNA in wild type but not *pYEF3:RPN4* cells. Fig. S2 shows that *Hsf1_Δ1-147_* expression activates the HSR and increases levels of Hsp104 and Sis1. Fig. S3 shows that folding stressors cause aggregation and recruitment of heat shock proteins. Fig. S4 shows the levels of unfolded protein response activity under proteolytic or folding stressors. Fig. S5 shows the levels and localization of short-lived proteins Nce103 and Ctk1 under folding and proteolytic stressors.
